# Rapid Detection of Animal-Derived Components in Plant-Based Meat Alternatives Using Recombinase Polymerase Amplification

**DOI:** 10.3390/foods14233992

**Published:** 2025-11-21

**Authors:** Yifei Sun, Han Li, Tianqi Ma, Tingting Xie, Yuqin Ni, Yu Chen, Xinya Chen, Wenke Ding, Zhuqing Xing

**Affiliations:** 1 School of Public Health, Tianjin University of Traditional Chinese Medicine, Tianjin 301617, China; 2 Institute of Neuroscience, Soochow University, Suzhou 215123, China

**Keywords:** plant-based meat alternatives, animal-derived components, DNA extraction methods, recombinant polymerase amplification

## Abstract

Plant-based meat alternatives (PMAs), as an emerging food category gaining increasing popularity, face potential food safety risks and ethical concerns for vegetarians due to the illegal adulteration of animal-derived components. To address these challenges and enhance regulatory oversight, the development of a rapid, sensitive, and highly specific detection method is essential. In this study, five DNA extraction methods were evaluated and optimized to identify the most effective approach for PMA products. The optimal conditions were determined to be 60 mmol/L NaCl, 10 mmol/L Tris HCl, and a centrifugation speed of 12,000× *g*. Additionally, specific primers targeting four common animal-derived adulterants, namely pork, chicken, duck, and beef, were designed and screened for targeted amplification. To establish a rapid and visually interpretable detection system, the recombinant polymerase amplification conditions were optimized. The final protocol used 0.4 µmol/L primer and isothermal amplification at 39 °C for 25 min, with the incorporation of SYBR Green I dye enabling the rapid and specific visualization of animal-derived DNA. This optimized method is characterized by its simplicity, sensitivity (capable of detecting beef-derived components as low as 0.0514% *w*/*w*), and rapidity, significantly reducing detection time and providing a reliable tool for the identification of animal-derived adulteration in PMA products.

## 1. Introduction

Plant-based meat alternatives (PMAs) are produced from vegetable proteins and are designed to mimic the flavor and appearance of real meat [1,2]. In recent years, with social progress and growing public health awareness, shifts in consumer preferences have emerged. In this context, plant-based meat alternatives (PMAs) have gained increasing attention as emerging substitutes to conventional meat, meeting evolving consumer demands [3,4]. However, driven by high profits, some producers illegally adulterate PMA products with animal-derived ingredients to obtain a more authentic flavor and texture [5]. This practice contradicts the original intentions of vegetarians in choosing PMAs while also undermining market standardization and posing potential food safety risks [6]. Therefore, rapid detection methods for animal-derived components in PMAs are not only crucial in verifying their authenticity but also play an indispensable role in import/export inspection and mutual recognition in international trade, having far-reaching significance in safeguarding consumer rights and ensuring food safety [7]. To date, domestic and foreign scholars have advanced research on the analysis of animal-derived components, with identification techniques trending toward increased diversification. Among them, nucleic acid-based molecular biology and protein-based immunoassay have emerged as standard methods [8,9]. In previous research conducted in our laboratory, processed meat analogs were found to exhibit unique peptide sequences after in vitro digestion, which can be used as biorecognition tags [10]. Moreover, although practical, protein-based assays are lengthy and costly and have a relatively narrow scope of application [11]. In contrast, polymerase chain reaction (PCR) based on DNA sequence specificity is primarily used in meat composition identification. However, this technique is limited by its harsh experimental conditions, a reliance on equipment such as thermocyclers, and the requirement for multiple steps, including nucleic acid extraction, PCR amplification, and electrophoresis, to obtain accurate results [12].

Recombinase polymerase amplification (RPA), developed by Twist Dx in the UK, is a revolutionary isothermal nucleic acid amplification method. It does not rely on a thermocycler and can efficiently complete the amplification reaction under constant temperature conditions [13]. Compared with other thermostatic amplification techniques, RPA has multiple advantages, such as lower cost, easy operation, high specificity, and shorter reaction time. Researchers regard it as a potential PCR alternative, bringing a significant breakthrough in nucleic acid detection with broad application potential [14]. However, the application of RPA for identifying PMA products is still in the early stages and needs further exploration and development. Currently, the detection of RPA-amplified products mainly relies on electrophoresis [15], the use of fluorescent dyes [16], and immunological methods [17]. Among these methods, electrophoresis requires a relatively long detection time, which diminishes the inherent efficiency and convenience of RPA. In this study, we developed a rapid, sensitive, and specific method for the detection of animal-derived ingredients in plant-based meat products by combining the RPA technology with SYBR Green I dye. We overcame the limitations of large-scale experimental instruments in field testing and enabled instant result visualization, thus improving the convenience and accuracy of testing. At the same time, we optimized the DNA extraction conditions for PMAs, reducing the DNA extraction time to accelerate research on the identification of authentic PMAs.

## 2. Materials and Methods

### 2.1. Materials

Plant-based chicken (ZWC, Bifei Ai Food Co., Ltd., Lu’an, China), plant-based pork (ZWP, Meikang Co., Ltd., Tianjin, China), and plant-based beef (ZWB, Cargill, Inc., Minneapolis, MN, USA) were obtained from local markets. Additionally, four types of real meat were acquired: pork (ZRP, Chia Tai Food Enterprise Co., Ltd., Shanghai, China), beef (ZRB, Mubiao Beef Industry Co., Ltd., Shanxi, China), chicken (ZRC, Cargill, Inc., Minneapolis, MN, USA), and duck (ZRD, Chia Tai Food Enterprise Co., Ltd., Shanghai, China) [18]. All reagents used in the study were of analytical grade.

### 2.2. Pretreatment Methods on DNA Extraction from PMAs

To investigate the effects of different pretreatment methods on DNA extraction from PMAs, we evaluated two pretreatment methods before DNA extraction. The optimal pretreatment method was determined based on the results obtained by UV spectrophotometry [19]. In pretreatment method 1, the sample was freeze-dried in pieces using a vacuum freeze dryer (FD-1A-50, Boyikang Experimental Instrument Co., Ltd., Beijing, China) and then ground with a sterile spoon. For pretreatment method 2, the frozen sample was thawed naturally at room temperature, cut into pieces, and soaked in a buffer solution (pH 8.0) consisting of 10 mmol/L Tris-HCl and 1 mmol/L EDTA for 3–6 h. After drainage, the samples were homogenized using a handheld homogenizer at 25,000 rpm for 30 s to form a slurry [13].

### 2.3. Optimizing the Extraction of Genomic DNA from PMAs

**Figure 1:**
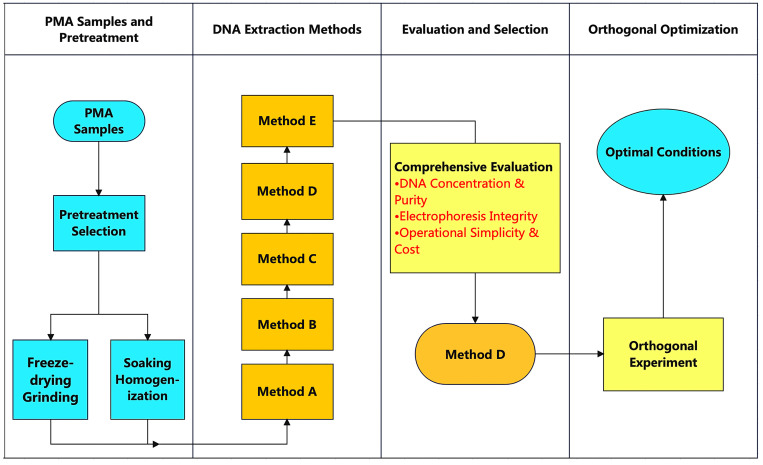
Schematic diagram of the systematic screening process for DNA extraction methods from plant-based meat alternatives (PMAs).

The overall strategy for evaluating and selecting the optimal DNA extraction method is depicted in Figure 1.

Three plant-based meat alternatives (ZWC, ZWP, and ZWB) were employed to evaluate the efficacy of different DNA extraction methods. The samples were thoroughly pretreated, and the following five methods were used to extract genomic DNA. The effectiveness of these methods was evaluated based on the concentration and purity of the extracted DNA, leading to the selection of the optimal DNA extraction method. Furthermore, the test factors were optimized through orthogonal experiments to determine the best method for extracting genomic DNA from PMAs.

#### 2.3.1. Method A

DNA extraction was carried out using a modified protocol as described by Xia, Y [20]. Specifically, 400 mg of the sample was weighed and transferred to a 1.5 mL sterile centrifuge tube, to which 1 mL of digestion buffer (containing 100 mmol/L NaCl, 10 mmol/L EDTA, 10 mmol/L Tris-HCl, 0.5% SDS, and 0.1 mg/mL protease K) was added. The mixture was vortexed for 10 s using a vortex mixer (VORTEX 3, IKA-Werke GmbH & Co. KG, Staufen, Germany) and incubated at 65 °C for 10 min. After centrifugation at 14,000× *g* for 2 min, the supernatant was collected and stored at −20 °C.

#### 2.3.2. Method B

DNA extraction was performed following a protocol adapted from Lee, G.Y [21] with minor modifications to optimize yield from PMAs samples. 50 mg of the sample was weighed and transferred to a 1.5 mL sterile centrifuge tube. Then, 400 µL of lysis buffer (containing 10 mmol/L Tris-HCl, 2 mmol/L EDTA, and 0.4 mmol/L NaCl) and 40 µL of 20% (*w*/*v*) SDS were added, and the mixture was vortexed strongly. Subsequently, 5 µL of 20 mg/mL protease K was added, and the tube was incubated at 65 °C for 1 h. Afterward, 300 µL of 6 mmol/L NaCl was added to the mixture, which was vortexed for 30 s. The mixture was centrifuged at 10,000× *g* for 30 min, and the supernatant was transferred to a sterile centrifuge tube. An equal volume of isopropanol was added to the supernatant, and the mixture was mixed by shaking. The tube was incubated at −20 °C for 10 min, followed by centrifugation at 16,000× *g* and 4 °C for 20 min. The supernatant was removed, and the precipitate was air-dried. The dried precipitate was dissolved in 100 µL of sterile water and stored at −20 °C.

#### 2.3.3. Method C

A modified protocol from Wendin, K [22] was used for DNA extraction. 100 mg of the sample was weighed and transferred to a 1.5 mL sterile centrifuge tube. Then, 1 mL of SDS extraction buffer (containing 2% SDS, 150 mmol/L NaCl, 50 mmol/L Tris-HCl, and 50 mmol/L EDTA) preheated to 65 °C and 10 µL of 10 mg/mL protease K were added. The tube was placed in a water bath at 65 °C for 1 h, with the tube being inverted every 10 min. The mixture was centrifuged at 12,000× *g* for 10 min and combined with chloroform/isoamyl alcohol (24:1, *v*/*v*) to extract the upper solution, which was repeated three times. To the upper aqueous phase, one-tenth volume of 3 mol/L potassium acetate (pH 5.5) and twice the volume of pre-cooled (−20 °C) 95% isopropanol solution were added, and the mixture was centrifuged at 15,000× *g* for 10 min. The pellet was washed twice with an ethanol solution and air-dried for 5 min. Finally, the dried DNA was redissolved in 200 µL of sterile water and stored at −20 °C.

#### 2.3.4. Method D

DNA was extracted according to a modified version of the protocol by Xia, Y [20], approximately 400 mg of the milled sample was weighed and transferred to a 1.5 mL sterile centrifuge tube. To the tube, 1 mL of rapid extraction solution (containing 50 mmol/L NaCl and 10 mmol/L Tris-HCl) was added, the mixture was vortexed for 10 s and then centrifuged at 14,000× *g* for 3 min, and the supernatant was stored at −20 °C.

#### 2.3.5. Method E

DNA extraction was performed using the Tigen DP 326 kit (DP326, Tiangen Biotech Co., Ltd., Beijing, China), following the product instructions for the reaction.

### 2.4. Determination of Genomic DNA Concentration and Purity

The purity of the sample DNA was assessed using a UV–visible spectrophotometer (P9, Shanghai Meipuda Instrument Co., Ltd., Shanghai, China) by calculating the *A*_260nm_/*A*_280nm_ ratio. The concentration of DNA extract was calculated using the following equation [23]:

*C*
(ng/μL) = 50 ng/μL × *A*_260nm_ × DF (1)

where *C* represents the DNA concentration, 50 ng/μL is the standard conversion factor for double-stranded DNA concentration per absorbance unit at 260 nm (*A*_260nm_), and DF represents the dilution factor.

### 2.5. Establishment of Adulteration Models and Evaluation of DNA Extraction Methods

#### 2.5.1. Establishment of Adulteration Models

Adulteration models were established with a 1:1 ratio between animal meat and the corresponding PMAs, except for duck meat, which was adulterated into plant-based chicken meat. To simulate the high-temperature processing typical of PMA production, DNA from fresh animal meat was thermally degraded in a boiling water bath for 3 min. For the initial comparative evaluation of the five DNA extraction methods, a 1:1 (*w*/*w*) adulteration model was used. To further determine the sensitivity of the optimized DNA extraction method, additional models were established according to the ratios of 1:1, 1:5, 1:10, and 1:20 for animal meat and PMAs.

#### 2.5.2. DNA Extraction and PCR Analysis

DNA was extracted from the 1:1 model samples using each of the five methods detailed in Section 2.3.1, Section 2.3.2, Section 2.3.3, Section 2.3.4 and Section 2.3.5. DNA from the gradient adulteration models was extracted using the optimized method determined in Section 2.5.3.

Given that PMAs are highly processed foods with inherently low DNA quality, direct electrophoretic analysis of the extracted DNA demonstrated suboptimal efficacy. Therefore, the effect of DNA extraction was evaluated by PCR. The PCR mixture was prepared according to Supplementary Table S1. Amplification was carried out in a PCR thermal cycler (TC1000-G, Beijing Dalong Xingchuang Experimental Instrument Co., Ltd., Beijing, China) using the universal primers listed in Supplementary Table S2. The PCR cycle parameters were set as follows: initial denaturation at 95 °C for 3 min; 35 cycles of denaturation at 95 °C for 30 s, annealing at 65 °C for 30 s, and extension at 72 °C for 30 s; followed by a final extension at 72 °C for 3 min, with subsequent storage at 4 °C.

The PCR products were then analyzed by agarose gel electrophoresis using the BIO-RAD electrophoresis system (BIO-RAD, Bio-Rad Laboratories, Inc., Hercules, CA, USA). A 2% agarose gel was prepared with nucleic acid stain (Tiangen Biotech Co., Ltd., Beijing, China) and 1× TBE as the electrophoresis buffer. A DNA marker and 3 µL of PCR-amplified DNA extract were mixed with 2 µL of loading buffer (Merck Life Science Biotech Co., Ltd., Darmstadt, Germany) and then loaded into the gel wells. Electrophoresis was conducted at 100 V for 30 min. After electrophoresis, the gel was imaged and the resulting electropherograms were used to evaluate the integrity of the extracted DNA.

#### 2.5.3. Optimization of the Genomic DNA Extraction Method

Based on the experimental results, method D was determined to be the optimal DNA extraction method for PMA products. A three-factor, three-level orthogonal test was conducted on the reagent ratio and centrifugation speed of method D (Table 1) further determine the optimal parameters for extracting genomic DNA from PMAs.

The limit of detection (LoD) expressed as a weight/weight percentage (% *w*/*w*) was calculated from the concentration-based LoD using the following equation:

LoD (% *w*/*w*) = [(C_LoD × V)/(DNA_content × m)] × 100% (2)

where C_LoD represents the minimum detectable DNA concentration (ng/μL), V is the final volume of the DNA extraction solution (μL), DNA_content is the mass fraction of DNA in animal muscle tissue, for which a commonly used estimate of 0.005 (0.5% *w*/*w*) was applied [24], and m is the mass of the PMA sample (mg) used for DNA extraction.

### 2.6. Determination of an Optimized DNA Extraction Method in an Adulteration Model

The optimized extraction method, as determined in Section 2.5.3, was used to prepare DNA extraction solutions from the standard and simulated adulterated PMAs described in Section 2.5.1. The applicability of this optimized method was evaluated by comparing the purity and concentration of the resulting genomic DNA solutions as described in Section 2.4.

### 2.7. Recombinase Polymerase Amplification

#### 2.7.1. Design of RPA Primers

The mitochondrial DNA genome sequences of pig, chicken, duck, and beef were obtained from GenBank, and multiple sequence comparisons were conducted to screen out specific fragments of animal origin. RPA primers were designed for the forward and reverse regions of specific fragments from each type of animal meat. The specific design principles are as follows: primer length, 30–35 nucleotides; GC content, 30–70%; Tm value, 70 ± 10 °C; the 5′ end of the primer should start with G as much as possible, avoiding consecutive C residues, and the 3′ end should end with G or C; and the amplicon length should be 150–300 bp, not exceeding 500 bp [25]. All primers detailed in Table 2 were synthesized by Suzhou Jinwei Zhi Biotechnology Co.

#### 2.7.2. RPA Method

The RPA reaction was performed according to the product specifications, with a total volume of 50 µL for the amplification system (Supplementary Table S3). To detect the possible adulteration of four specific animal-derived DNA (from chicken, beef, pork, and duck) in PMAs, the corresponding species-specific primer pairs (as listed in Table 2) were added to the reaction system. Subsequently, B buffer was added to the cap of the reaction tube, which was mixed well by inverting the tube 8–10 times, and the test tube containing the reaction materials was incubated in a water bath at 37–39 °C for 30 min. After the reaction was completed, 50 µL of an extraction solution consisting of Tris-saturated phenol, chloroform, and isoamyl alcohol in a ratio of 25:24:1 was added to 50 µL of the reaction product for purification. The mixture was thoroughly mixed by inverting and then centrifuged at 16,000× *g* for 5 min.

To obtain the ideal primer pair for the RPA reaction, the DNA extracts were amplified using three PCR primer pairs, ensuring that the species origin of each DNA extract matched that targeted by the RPA primers. After the reaction, the optimal primer pairs for RPA of the four types of animal meat (chicken, beef, pork, and duck) were determined using 2% agarose gel electrophoresis.

#### 2.7.3. Optimization of RPA Reaction Conditions

To establish the optimal reaction conditions, key parameters including primer concentration and incubation temperature were optimized using genomic DNA extracted from the 1:20 animal meat-PMA adulteration models with Method D. To correspond with the animal-derived components (at a concentration of 10 µmmol/L), 1.0, 2.0, and 3.0 µL of forward and reverse primers were added to achieve final concentrations of 0.2, 0.4, and 0.6 µmmol/L, respectively, in the 50 µL RPA reaction system. The RPA reaction was initiated at 37 °C, and upon completion, the bands were analyzed using agarose gel electrophoresis to determine the optimal concentrations of forward and reverse primers for the RPA reaction.

Subsequently, the optimal reaction temperature was investigated. The samples and DNA extract were prepared following the previously described procedure. Four temperatures (35, 37, 39, and 41 °C) were tested using 2% agarose gel electrophoresis.

#### 2.7.4. Visual Assay for RPA Reactions Design

Briefly, 2 µL of SYBR Green I dye (Biosharp Biotech Co., Ltd., Hefei, China) was added to 25 µL of the purified amplification product, and the fluorescence signal was observed under 365 nm UV irradiation using a gel imaging analyzer.

##### Effect of Different Factors

The impact of reaction time on the assay’s performance was first evaluated. Using DNA from the four simulated adulteration models of standard chicken, beef, pork, and duck (with a 1:20 ratio of animal meat to PMAs), and five reaction times (15, 20, 25, 30, and 35 min) were tested.

The specificity of the assay was then confirmed against a panel of templates. Multiple groups were set up with the following templates: a positive group using DNA from four simulated adulteration models of standard chicken, beef, pork, and duck (with a 1:20 ratio of animal meat to PMAs); a negative group using soybean DNA; and a blank group using an equal volume of ddH_2_O.

To assess the robustness of the method, its stability over time was tested. DNA was extracted from the beef adulterated model and soybean samples; the extracted DNA was then stored at −20 °C. The RPA assay was repeated after 1, 2, and 3 days to check for any false positives.

Finally, the sensitivity of the visual detection was determined by testing a series of DNA concentrations. Using DNA from the beef adulteration model, five DNA concentrations (300, 30, 3, 0.3, and 0.03 ng/µL) were prepared in sterile water, along with the negative control, using a 10-fold gradient dilution.

### 2.8. Data Processing

All data were expressed as the mean ± standard deviation of three replicate measurements. The data were summarized and calculated using Excel 2019. Quantification of the fluorescence signal (G/R ratio) from the gel images was performed using Fiji. Comparisons of different pretreatments were performed using non-parametric tests (Wilcoxon signed-rank test), while the remaining experimental results were analyzed using a one-way ANOVA followed by Duncan’s multiple range test conducted with SPSS 27.0 software. Differences were considered statistically significant at *p* < 0.05.

The following equations were used for the calculation of method-validation metrics:

The repeatability and intermediate precision were expressed as the coefficient of variation (CV):

CV (%) = (Standard Deviation / Mean) × 100%(3)

Diagnostic sensitivity (Se), specificity (Sp), positive predictive value (PPV), and negative predictive value (NPV) were calculated as follows:

Se (%) = [TP/(TP + FN)] × 100%(4)

Sp (%) = [TN/(TN + FP)] × 100%(5)

PPV (%) = [TP/(TP + FP)] × 100%(6)

NPV (%) = [TN/(TN + FN)] × 100%(7)

where TP represents true positives, FN false negatives, TN true negatives, and FP false positives. Confidence intervals (95%) for these proportion metrics were calculated using the Clopper-Pearson (exact) method.

## 3. Results

### 3.1. Purity and Concentration of the Five DNA Extraction Methods

All methods were capable of successfully extracting DNA, yet significant differences existed in their efficacy (Supplementary Table S4). The concentration of PMA DNA obtained using the five extraction methods generally exceeded 240 ng/µL. Method E extracted DNA samples of PMAs that exhibited *A*_260nm_/*A*_280nm_ ratios of 1.734 ± 0.009, 1.664 ± 0.004, and 1.685 ± 0.001 (Supplementary Table S5). These ratios were close to 1.7, suggesting a relatively high DNA purity. Conversely, methods C and D extracted genomic DNA with *A*_280nm_ ratios generally above 1.60, indicating potential slight contamination by proteins and other impurities, although the overall purity remained acceptable. However, methods A and B extracted DNA with *A*_260nm_/*A*_280nm_ ratios below 1.60, suggesting higher levels of impurities, such as proteins, resulting in relatively lower purity. As shown in Supplementary Table S6, there were significant differences (*p* < 0.05) in the purity of DNA extracted using each method, except for ZWP extracts, where methods B and D showed no significant differences (*p* > 0.05). Notably, method E achieved the highest average *A*_260nm_/*A*_280nm_ ratio, while method A had the lowest. The average purity of the remaining three methods was comparable. As shown in Supplementary Table S5, methods B–E yielded ZWP DNA extraction concentrations with no significant differences (*p* > 0.05). Upon comparative analysis, no statistically significant difference was observed in the DNA extract concentration from ZWC between methods A and B (*p* > 0.05); however, significant differences were observed among the other extraction methods (*p* < 0.05).

### 3.2. Agarose Gel Electrophoresis Detection Results of Five DNA Extraction Methods

Due to their functional limitations, UV spectrophotometers can provide information on DNA concentration and purity but cannot directly assess DNA integrity. To comprehensively evaluate the quality of extracted DNA, especially its potential degradation, this study used agarose gel electrophoresis for analysis. However, because PMAs are highly processed foods with low DNA content, they cannot be accurately measured using standard agarose gel electrophoresis. Therefore, this study used PCR to evaluate the effectiveness of DNA extraction.

**Figure 2:**
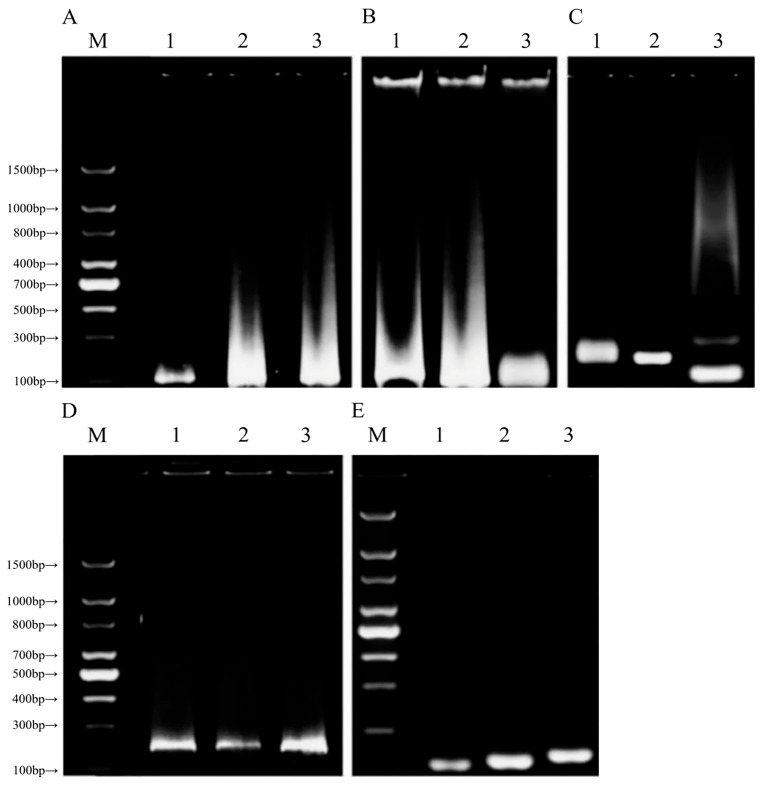
Agarose gel electrophoresis detection map of DNA extracted using five methods, (**A**) Method A, (**B**) Method B, (**C**) Method C, (**D**) Method D, (**E**) Method E (M: DNA ladder; 1: ZWC adulteration model; 2: ZWB adulteration model; and 3: ZWP adulteration model).

DNA extracted using method A showed partial degradation in the ZWC adulteration model (Figure 2A). The corresponding gel lanes exhibited unclear DNA bands, and no protein residues remained in the sample wells. DNA samples from the ZWP and ZWB adulteration models showed smearing and tailing, indicating significant degradation and low DNA integrity. In Figure 2B, brighter bands appear at the sample wells, suggesting slight protein contamination in the DNA extracted using method B. Lanes 1 and 2 display visible bands but show tailing, indicating that method B successfully extracted DNA from the ZWC and ZWB adulteration models, albeit with poor integrity and contamination. In Figure 2C, the bands show uniform brightness with slight, localized tailing, indicating good overall genomic DNA integrity with minimal degradation. Figure 2D shows clear and well-distributed DNA bands, along with some fluorescence diffusion, suggesting high purity with minimal contamination and partial degradation. In Figure 2E, the extracted DNA bands are sharp and bright, indicating high integrity, free from RNA contamination, and no interference from polysaccharides or other impurities. All methods extracted DNA from the samples, but methods A and B showed relatively poor performance. Methods D and E successfully amplified sharp DNA bands in all test samples, demonstrating excellent integrity. These high-quality DNA extractions were suitable for subsequent RPA reactions.

After the comprehensive consideration of factors such as concentration, purity results, ease of operation, and economic cost, method D was ultimately selected as the basis for subsequent experiments.

### 3.3. Purity and Concentration of DNA Extracted Using Different Pretreatment Methods

To investigate the effect of different pretreatment methods on the purity and concentration of genomic DNA extracts, we used two distinct pretreatment methods alongside DNA extraction method D to extract genomic DNA (Supplementary Table S6). The *A*_260nm_/*A*_280nm_ ratio of genomic DNA extracted using both pretreatment methods exceeded 1.640, with concentrations above 290 ng/µL, meeting the requirements for molecular biology applications. Compared with pretreatment method 1, pretreatment method 2 slightly improved the purity and concentration of the obtained DNA extracts due to the prolonged immersion and high-speed homogenization of the samples. However, the genomic DNA remained contaminated by proteins. The DNA purity and concentration of the two pretreatment methods with PMAs showed no significant difference (*p* > 0.05). In subsequent primer validation experiments, the templates used in method 2 failed to amplify the target bands in some cases, so method 1 was selected for the pretreatment process.

### 3.4. Optimization of DNA Extraction Method by Orthogonal Experiments

An orthogonal experimental design with three factors and levels totaling nine experimental conditions was established to simulate the PMA adulteration model (Supplementary Table S7). The calculation of DNA purity (*A*_260nm_/*A*_280nm_) and range analysis revealed the influence patterns of the factors, as summarized in Supplementary Table S7. The range values determined the order of influence on the DNA extraction purity: *R*_C_ = 0.110 > *R*_A_ = 0.090 > *R*_B_ = 0.084. Centrifugation speed exerted the greatest effect on DNA purity, followed by NaCl concentration and Tris-HCl concentration. NaCl concentration had a relatively higher influence on DNA purity than Tris-HCl concentration.

The analysis identified the optimal levels for DNA extraction from plant-based meat as A3, B2, and C3. Further validation experiments using 10 mmol/L Tris-HCl, 60 mmol/L NaCl, and a centrifugation speed of 12,000× *g* resulted in the highest DNA purity of 1.675 ± 0.003. This purity surpassed the results obtained in all orthogonal experimental groups.

### 3.5. Efficacy of an Optimized DNA Extraction Method in Adulterated Products

The experiment used an optimized method to extract genomic DNA from simulated adulterated models through rapid extraction. The DNA purity and concentration were measured using UV spectrophotometry, and the results are summarized in Table 3. When the ratio of animal meat to PMAs reached 1:20, method D produced DNA with a purity of 1.649 ± 0.002 and a concentration of 277.8 ± 5.8 ng/µL, surpassing the results from the other three adulteration ratios. Protein contamination affected DNA extracts to varying degrees across the four adulteration ratios, but the *A*_260nm_/*A*_280nm_ ratios consistently remained above 1.54, and the concentrations always exceeded 250 ng/µL. These measurements met the molecular biology experiment standards and confirmed successful RPA. Method D effectively extracted DNA, demonstrating superior suitability.

### 3.6. Screening Results of Optimal Primer Pairs for RPA Reactions

**Figure 3:**
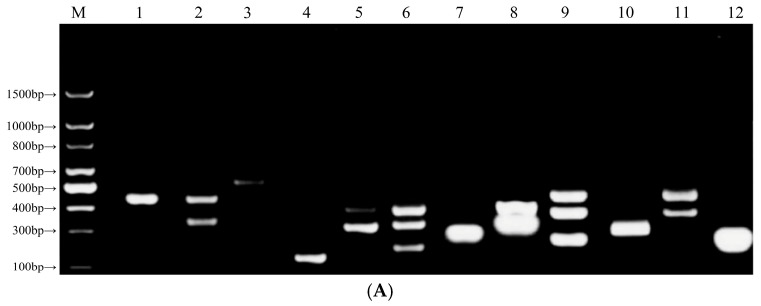

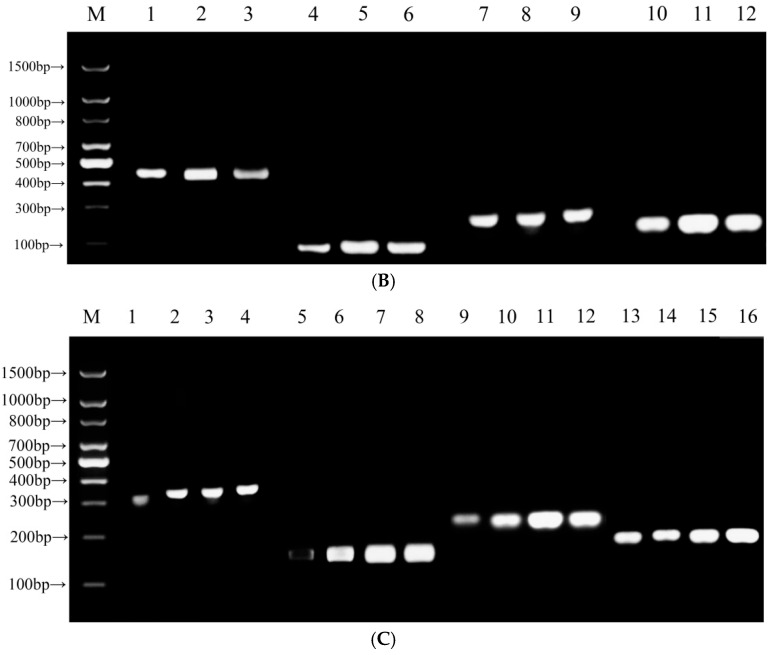
Agarose gel electrophoresis detection maps for RPA optimization (**A**) optimal primer pairs (M: DNA ladder; 1: JI1F/JI1R; 2: JI2F/JI2R; 3: JI3F/JI3R; 4: NIU1F/NIU1R; 5: NIU2F/NIU2R; 6: NIU3F/NIU3R; 7: ZHU1F/ZHU1R; 8: ZHU2F/ZHU2R; 9: ZHU3F/ZHU3R; 10: YA1F/YA1R; 11: YA2F/YA2R; and 12: YA3F/YA3R), and (**B**) optimal primer concentrations (M: DNA ladder; 1: ZWC-ZRC 0.2 µM; 2: ZWC-ZRC 0.4 µM; 3: ZWC-ZRC 0.6 µM; 4: ZWB-ZRB 0.2 µM; 5: ZWB-ZRB 0.4 µM; 6: ZWB-ZRB 0.6 µM; 7: ZWP-ZRP 0.2 µM; 8: ZWP-ZRP 0.4 µM; 9: ZWP-ZRP 0.6 µM; 10: ZWC-ZRD 0.2 µM; 11: ZWC-ZRD 0.4 µM; and 12: ZWC-ZRD 0.6 µM), and (**C**) optimal reaction temperature conditions (M: 100 bp DNA ladder; 1: ZWC-ZRC 35 °C; 2: ZWC-ZRC 37 °C; 3: ZWC-ZRC 39 °C; 4: ZWC-ZRC 41 °C; 5: ZWB-ZRB 35 °C; 6: ZWB-ZRB 37 °C; 7: ZWB-ZRB 39 °C; 8: ZWB-ZRB 41 °C; 9: ZWP-ZRP 35 °C; 10: ZWP-ZRP 37 °C; 11: ZWP-ZRP 39 °C; 12: ZWP-ZRP 41 °C; 13: ZWC-ZRD 35 °C; 14: ZWC-ZRD 37 °C; and 15: ZWC-ZRD 39 °C; 16: ZWC-ZRD 41 °C).

To achieve the best detection results, the experiment identified the optimal primer pairs for RPA reactions. This process involved the extraction of genomic DNA from chicken, beef, pork, and duck meat and its amplification using PCR systems with the corresponding primers. The analysis of the amplification results relied on agarose gel electrophoresis (Figure 3A). The analysis confirmed that primers JI1F/R, NIU1F/R, ZHU1F/R, and YA1F/R exhibited high specificity, strong target selectivity, and fragment sizes consistent with the desired final products. In contrast, other primer pairs showed lower amplification specificity, with faint nonspecific bands appearing above the main bands. This evaluation confirmed JI1F/R, NIU1F/R, ZHU1F/R, and YA1F/R as the optimal primer pairs for subsequent RPA detection reactions.

### 3.7. Optimization of Primer Concentration for RPA Reactions

The final primer concentrations of 0.2, 0.4, and 0.6 µmol/L for each reaction system were tested. The lanes corresponding to the 0.4 µmol/L primer concentration consistently produced a single band with high signal intensity, clear definition, and good integrity, aligning with the desired fragment size across all four adulteration models (Figure 3B). These bands also exhibited significantly greater brightness than those in the lanes for the 0.2 and 0.6 µmol/L concentrations. The analysis confirmed that adding 2.0 µL of forward and reverse primers to the 50 µL RPA reaction system (to achieve a final primer concentration of 0.4 µmol/L) delivered the best amplification efficiency. In addition, the study established this primer volume as the standard protocol for subsequent RPA experiments.

### 3.8. Optimization of RPA Reaction Temperature

Reaction temperature critically influenced the stability and accuracy of the experiment, as extreme temperatures negatively affected the results. The experiment evaluated four temperature gradients (35, 37, 39, and 41 °C) by conducting amplification in a thermostatic water bath. The results were analyzed using agarose gel electrophoresis after amplification. The chicken and beef adulteration models did not produce clear and complete bands at 35 °C (Figure 3C). In contrast, all other adulteration models generated specific bands matching the desired fragment size within the 35–41 °C range. Amplification at 39 °C and 41 °C demonstrated superior specificity for the target bands. Considering the potential negative effects of higher temperatures on enzyme activity, the study identified 39 °C as the optimal amplification temperature and applied this condition in subsequent experiments.

### 3.9. Optimization of RPA Reaction Time

**Figure 4:**
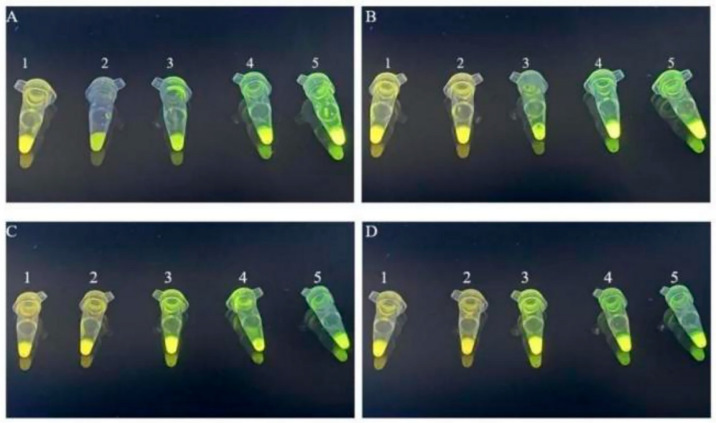
Fluorescence reaction at the optimized RPA reaction time using SYBR Green I (**A**): ZWC adulteration model; (**B**): ZWB adulteration model; (**C**): ZWP adulteration model; and (**D**): ZWD adulteration model—1: 15 min; 2: 20 min; 3: 25 min; 4: 30 min; and 5: 35 min).

The experiment examined the influence of RPA reaction time on the amplification results and efficiency by testing five durations, i.e., 15, 20, 25, 30, and 35 min, using the RPA and SYBR Green I reaction system with optimized primer concentration and reaction temperature. After 15 min, the RPA for all four adulteration models had not reached the plateau phase (Figure 4). SYBR Green I emitted weak yellow fluorescence due to insufficient binding with double-stranded DNA, which was quantitatively supported by G/R ratios approaching 1.0 (e.g., A1: 0.9948 ± 0.0121, B1: 0.9882 ± 0.0142, C1: 0.9833 ± 0.0137, D1: 0.9655 ± 0.0158) (Supplementary Table S8). After 20 min, the chicken and beef adulteration models reached the plateau phase; however, the SYBR Green I fluorescence was weak, indicating incomplete DNA amplification, as reflected in the moderate G/R ratios (A2: 1.1219 ± 0.0065, B2: 1.0974 ± 0.0104) that were significantly above 1.0 yet not at maximum levels. The pork and duck adulteration models still failed to reach the plateau phase, with G/R ratios remaining near 1.0 (C2: 0.9957 ± 0.0123, D2: 0.9551 ± 0.0146). After 25, 30, and 35 min, all four adulteration models displayed visible green fluorescence, indicating the successful binding of SYBR Green I to adequately amplified double-stranded DNA, which was corroborated by consistently elevated G/R ratios significantly exceeding 1.0 across all models. To balance time efficiency and amplification performance, 25 min was chosen as the optimal RPA reaction duration, as it represented the earliest time point where all models demonstrated both statistically significant fluorescence amplification (G/R > 1.0) and stable signal intensity.

### 3.10. Specificity of RPA Reactions

**Figure 5:**
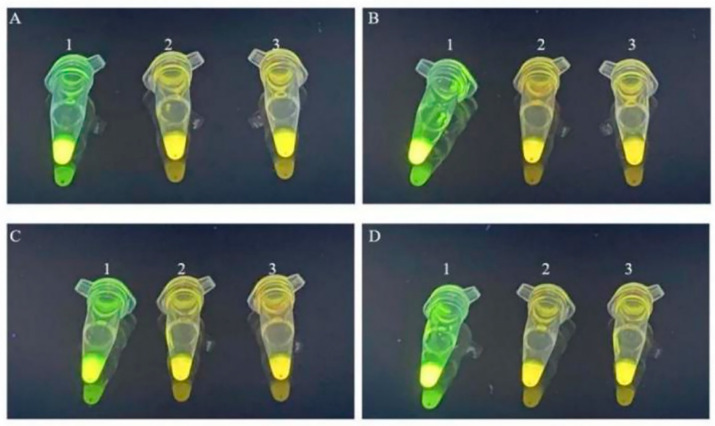
Fluorescence reaction of RPA-specific detection using SYBR Green I (**A**): ZRC adulteration model; (**B**): ZRB adulteration model; (**C**): ZRP adulteration model; and (**D**): ZRD adulteration model—1: active control; 2: negative control; and 3: blank control).

The experiment assessed the specificity of RPA primers for detecting animal-derived components by setting up positive, negative, and blank control groups under optimized RPA reaction parameters. Fluorescence signals appeared exclusively in the positive control group, with neither the blank control nor the negative control groups showing any fluorescence (Figure 5). This observation was quantitatively confirmed, as significantly higher G/R ratios (*p* < 0.05) were measured exclusively in the positive controls compared to all negative and blank controls (Supplementary Table S9). The RPA primers effectively amplified animal-derived DNA without reacting with plant-derived DNA, demonstrating their excellent specificity.

### 3.11. Stability of RPA Reactions

The experiment used the cattle adulteration model to verify the stability of RPA (Supplementary Figure S1). Tubes containing animal-derived components consistently showed positive reactions, with G/R ratios significantly higher (mean = 1.0165, *p* < 0.05) than those of negative controls (mean = 0.9598), as quantified in Supplementary Table S10. This confirms the effectiveness and consistency of the method in specifically detecting animal-derived components. The system amplified only animal-derived DNA without reacting with other genomic DNA, ensuring accurate detection results. Furthermore, the results remained consistent over repeated tests without any erroneous changes, demonstrating the robust stability of the RPA assay.

### 3.12. Sensitivity of RPA Reactions

**Figure 6:**
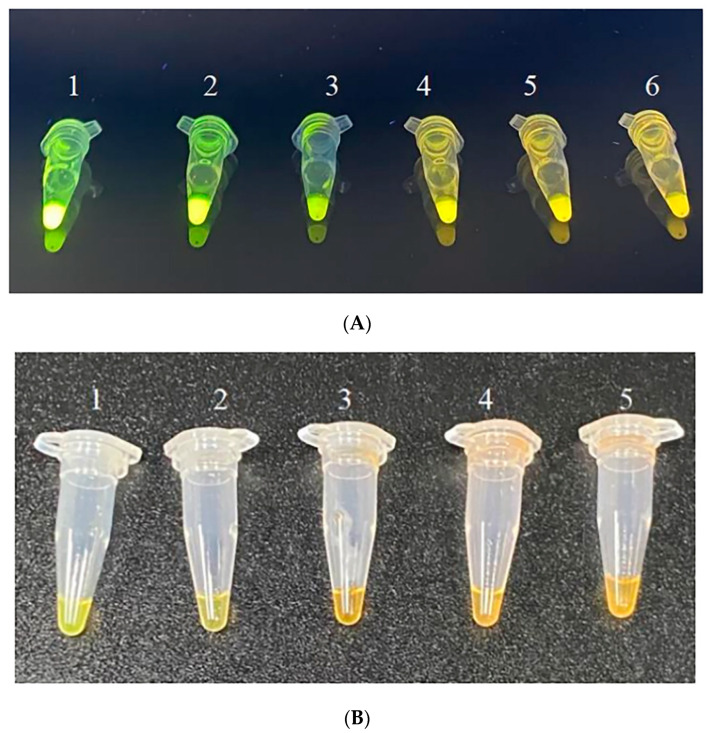
Sensitivity of RPA detection based on SYBR Green I (**A**) fluorescence reaction (1: 300 ng/µL; 2: 30 ng/µL; 3: 3 ng/µL; 4: 0.3 ng/µL; 5: 0.03 ng/µL; and 6: Blank control), and (**B**) chromaticity reaction (1: 300 ng/µL; 2: 30 ng/µL; 3: 3 ng/µL; 4: 0.3 ng/µL; and 5: 0.03 ng/µL).

The experiment used the beef adulteration model to test the sensitivity of the RPA reaction system by amplifying DNA solutions with five concentration gradients and the negative control. After reaction completion, SYBR Green I dye was added, and the fluorescence color changes were observed under 365 nm UV light using a gel imaging analyzer. Quantitative analysis (Supplementary Table S11) confirmed significantly higher G/R ratios (*p* < 0.05) at DNA concentrations ≥3 ng/µL (e.g., 1.2146 ± 0.0145 at 3 ng/µL), compared to concentrations below this threshold (≤0.9873), this approach identified the lowest detectable DNA template concentration for visual detection. In Figure 6A, microtubes numbered 1 to 6 correspond to DNA concentrations of 300, 30, 3, 0.3, 0.03, and 0 ng/µL (blank control), respectively. Groups with template DNA concentrations ≥3 ng/µL showed a distinct green fluorescence, while those with concentrations <3 ng/µL retained a yellow fluorescence without significant change. Therefore, the minimum detectable DNA concentration for the RPA system was 3 ng/µL. Under natural light, as quantified in Series B (Supplementary Table S11), groups with DNA concentrations ≥30 ng/µL (G/R ratio ≥ 1.0205) displayed noticeable green color changes visible to the naked eye (Figure 6B). While this sensitivity was slightly lower than the fluorescence observed under 365 nm UV light at concentrations ≥3 ng/µL, the use of natural light simplified the process by reducing equipment requirements and offering convenient, visually observable results.

The uncertainty of this visual LoD was quantified based on the variability in G/R ratios at the detection limit. With a relative standard uncertainty of 1.19%, the expanded uncertainty (k = 2) is 0.0012% (*w*/*w*). Thus, the visual detection limit is 0.0514% ± 0.0012% *w*/*w* (k = 2).

The highly sensitive LoD demonstrated here for beef (0.0514% ± 0.0012% *w*/*w*, k = 2) serves as a key performance indicator of the optimized DNA extraction method (Method D). This result quantitatively confirms that the rapid extraction protocol yields DNA of sufficient quality and quantity to support ultrasensitive detection in complex PMA matrices.

### 3.13. Analytical Performance of the RPA Assay

The diagnostic performance and precision of the RPA assay were rigorously evaluated using all controlled experiments conducted throughout this study. The method demonstrated excellent repeatability (1.19%) and intermediate precision (0.16%), alongside ideal diagnostic sensitivity and specificity (both 100%). A complete summary of all validation metrics, including calculated values with 95% confidence intervals, is provided in Supplementary Table S12.

## 4. Discussion

### 4.1. Effect of Different Pretreatment Methods on DNA Extraction

Molecular biology experiments require high precision, demanding specific techniques and accumulated experience. During DNA extraction, rapid operation, low-temperature environments (such as ice baths), and thorough grinding are the key factors that ensure successful mitochondrial DNA isolation. Among these, the grinding step plays a crucial role in influencing the purity and integrity of the DNA [26]. In this study, pretreatment method 1 involved the thorough grinding of freeze-dried samples, ensuring the successful extraction of adulterated model DNA. Although pretreatment method 2 achieved slightly higher DNA purity and concentration, the results did not significantly differ from those of method 1. Additionally, during the primer validation experiment, method 2 yielded unsatisfactory amplification results, possibly due to DNA degradation exacerbated by room-temperature thawing and prolonged soaking. Therefore, pretreatment method 1 was chosen for subsequent experiments.

Common DNA extraction methods include enzymatic digestion, cetyltrimethylammonium bromide (CTAB), magnetic bead, and phenol-chloroform extraction. However, enzymatic digestion, while capable of obtaining high-quality DNA, is time-consuming, incurs higher costs, and cannot avoid DNA degradation under high temperatures [27]. The CTAB method, though an excellent choice for plant DNA extraction, often requires prolonged soaking due to interference from plant polysaccharides [28]. Similar to method 2, this prolonged soaking increases the risk of contamination and results in low efficiency for animal sample extraction, making it unsuitable for detecting animal meat in plant-based meat products. Magnetic bead extraction and phenol/chloroform methods can yield highly purified DNA but involve complex and expensive equipment and reagents. Moreover, they may contaminate DNA with chemical reagents and are unsuitable for rapid on-site detection [29,30]. Therefore, the methods used in this study, particularly freeze-drying and grinding, offer significant advantages for fast, efficient, and low-cost DNA extraction.

### 4.2. DNA Extraction Methods for PMAs

The *A*_260nm_/*A*_280nm_ ratio is a key indicator for assessing DNA purity. A standard ratio ranges from 1.8 to 2.0, where higher values indicate potential RNA contamination and lower values suggest protein contamination in the DNA sample [31]. High levels of polyphenols and polysaccharides in the samples may lower the *A*_260nm_/*A*_280nm_ ratio [32]. The complex processing and high additive content of PMA products, particularly highly processed variants, often result in extracted DNA with reduced purity.

Notably, trace-level adulteration also leads to reduced DNA extraction purity, particularly in autoclaved or highly processed products. For example, in mixed pork–beef matrices containing 0.01% pork, DNA fragmentation (80–500 bp) and subsampling errors significantly increased technical variability (CV > 45%) and bias (>70%), highlighting the challenges of quantifying low-concentration DNA [33]. While such issues could be mitigated through DNA treatment or ddPCR optimization, the observed effects in this study were minimal, and thus, the experimental protocol remained unaltered.

DNA concentration measurements of ZWP showed no significant differences among the four extraction methods (methods B–E). This lack of variation may arise from differences in the processing characteristics of plant-based pork compared with plant-based beef or chicken. Additives and colorants in the samples likely restricted the practical efficiency of the DNA extraction methods, resulting in similar DNA concentrations.

Method E, which applied the commercial test kit, delivered the best DNA extraction performance but required extensive procedures, taking approximately 140 min per sample. This method incurred high costs and offered no substantial improvement in DNA purity compared with other methods. In contrast, method D extracted DNA within approximately 5 min using common reagents. This approach reduced reliance on specialized equipment, minimized costs, and simplified operations. Although the DNA obtained using method D remained incomplete and could lower the sensitivity of subsequent analyses, it significantly reduced the processing time. Considering the high sensitivity of the RPA-based adulteration detection method established in this study, using crude DNA extracts ensures substantial time savings and reduces extraction costs [34]. The critical role of template DNA in determining detection sensitivity is well-supported, as demonstrated in studies quantifying the relationship between template concentration and detection probability [35]. To optimize efficiency, subsequent experiments used method D for DNA extraction. When extracting DNA from adulterated samples of varying proportions using the optimal method, the obtained DNA purity and concentration were lower than those of PMA samples. This may be attributed to the heating process during the preparation of simulated adulterated samples, which further disrupted the DNA structure in the original PMA samples and resulted in certain losses.

### 4.3. RPA Reaction System for Detecting Adulteration in PMA Products

Meat adulteration, a serious form of food fraud, involves mixing other meat components into meat or meat products, making them indistinguishable by appearance and allowing producers to gain higher economic benefits. In recent years, meat adulteration has posed a significant threat to the healthy development of the meat industry. Thus, there is an urgent need for efficient and reliable adulteration detection methods [36]. Molecular biology methods for detecting meat adulteration often require specialized instruments that are rarely accessible for routine use, limiting their practicality for on-site rapid testing [37]. RPA, an innovative nucleic acid isothermal amplification technique, overcomes these limitations with its remarkable sensitivity and specificity [38], offering breakthroughs in rapid on-site detection [39].

RPA technology has already been applied in the adulteration detection of traditional meat products, enabling the rapid and sensitive identification of low-concentration adulterated meat. In a study by [40], RPA technology was applied to detect chicken adulteration in meat products. They used RPA primers targeting the chicken *Cytb* gene and optimized the system to improve specificity and shorten the reaction time. This method successfully detected chicken DNA, providing a sensitivity of 8 copies/µL within 60 min. Liu employed universal primers and specific crRNA to amplify DNA from multiple meat sources, using RPA to achieve a sensitivity of 10 pg/μL and complete multi-channel detection within 30 min, demonstrating the high specificity and sensitivity of RPA [41]. This aligned with our study, where we optimized RPA primers for animal DNA to minimize cross-reaction with plant-derived DNA and achieved a detection limit of 4.76% animal meat content in plant-based products. It is noteworthy that a previous study utilizing a combined RPA-CRISPR/Cas12a system for detecting goat milk powder adulteration reported a detection limit as low as 1% for the adulterant, which is lower than the result observed in our experiment [42]. Aside from inter-species and methodological differences, this discrepancy likely originates from the solid-state characteristics of the adulteration simulants used in our work. The difficulty in achieving complete homogenization in solid matrices can lead to particle effects and uneven distribution of the target analyte. Consequently, the effective local concentration presented for extraction and amplification may vary, potentially accounting for the observed difference in results. Additionally, our study optimized the system to a 25 min detection window, offering a significant time advantage.

To visually detect adulteration using RPA with an isothermal DNA amplification kit, SYBR Green I, a widely used and cost-effective fluorescent dye, was incorporated into the RPA products. The fluorescence signals were then observed using a gel imaging analyzer to confirm the presence of animal-derived amplification fragments in the reaction system. The detection process allowed for the visualization of adulteration under natural light with DNA concentrations of ≥30 ng/µL. Under 365 nm UV light, the fluorescence coloration appeared stronger and more distinct than the results observed with the 20× solution. This RPA-based visual detection method for PMA adulteration relied on genomic DNA extracted through simple procedures without purification. The minimum detectable DNA concentration in adulteration models reached 3 ng/µL.

Currently, several studies have integrated RPA technology with other instruments to achieve visualization and enhanced detection accuracy. RPA-based visualization is commonly performed using a surface-enhanced Raman scattering (SERS) sensor. In a previous study by [43], the combination of RPA and SERS signal detection was relatively fast (26 min), with high specificity and stability. Another study by Liu employed a two-color lateral flow immunochromatographic strip, in which functionalized gold nanoparticles were conjugated with probes targeting chicken and duck DNA, achieving a detection time of 20 min [44]. This mRPA system targeted mitochondrial DNA, offering strong stability and making it suitable for complex food samples. However, these methods require high-end equipment and involve complex procedures, which makes them less suitable for rapid on-site detection. Although the detection sensitivity of the method used in this study is at the nanogram level, which is slightly lower than the picogram-level sensitivity achieved by these methods, there is not a significant difference in practical application. Moreover, the simpler operational workflow provides distinct advantages in usability and accessibility.

In this experiment, we established a relatively rapid, sensitive, and highly stable measurement method well suited for use in various frontline market regulatory scenarios. However, the quantity or specific tissue source of the additives cannot be directly inferred through visual inspection. Furthermore, the detection of some animal-derived additive products may not be feasible due to the complexity of deep-processing techniques, high degree of DNA destruction, and low quantity of additives, which necessitate more sophisticated instrumental measurement methods.

The RPA-based visual detection method established in this study demonstrates excellent potential for the rapid screening of animal-derived adulteration in PMAs. It is important to note, however, that the assay’s performance was validated using simulated adulteration models. While the use of three distinct commercial PMA bases (plant-based chicken, pork, and beef) in this study supports its broad applicability, future work should include a formal validation involving a larger panel of commercial products from various brands and blinded spiking experiments. This will conclusively determine the diagnostic sensitivity and specificity of the method in real-world market surveillance scenarios.

### 4.4. Implications for More Aggressive Heat Treatments

It should be noted that this study utilized a boiling water bath to simulate thermal processing. While this approach effectively induced DNA fragmentation, even more aggressive treatments like frying could cause further degradation. The established positive correlation between heating intensity and DNA damage strongly suggests that such severe treatments would follow, and likely intensify, the observed pattern of reduced RPA efficiency [45]. Therefore, future work should explicitly validate this assay in products subjected to extreme conditions like frying. The strategies central to this method—short amplicon design and system optimization—nonetheless provide a robust foundation for detecting adulteration in a wide range of processed foods.

## 5. Conclusions

This study developed a rapid and visual method for detecting animal-derived components in plant-based meat alternatives (PMAs) by integrating recombinase polymerase amplification (RPA) with SYBR Green I dye. We first evaluated and optimized the DNA extraction process for PMAs, determining that a crude extraction method (using 60 mmol/L NaCl, 10 mmol/L Tris-HCl, and centrifugation at 12,000× *g*) following freeze-drying and grinding was optimal. This method efficiently produced DNA of sufficient quality while significantly simplifying operations and reducing costs.

Subsequently, specific RPA primers were designed for pork, chicken, duck, and beef components. The RPA reaction conditions were optimized to 39 °C for 25 min with a primer concentration of 0.4 µmol/L. The incorporation of SYBR Green I dye enabled direct visual detection of amplification products under UV light (365 nm), with a sensitivity of 0.0514% ± 0.0012%. The entire process, from DNA extraction to result interpretation, can be completed within approximately 35 min.

In conclusion, this study developed a rapid and visual detection method by integrating recombinase polymerase amplification (RPA) with SYBR Green I dye. This system offers simplicity, rapid response, and high specificity, enabling direct visualization of results for on-site preliminary screening. It is critical to underscore that positive outcomes obtained via this on-site assay are provisional and mandate subsequent confirmation utilizing established, gold-standard laboratory techniques, such as conventional PCR in conjunction with electrophoresis or mass spectrometry, to achieve definitive identification. As a powerful frontline tool, this innovative approach provides a convenient, instrumental-free solution for upholding the authenticity of PMA products, driving advancements in food authenticity technologies, and strengthening food safety regulatory frameworks.

## Figures and Tables

**Table 1:** DNA extraction orthogonal test factors and levels.

Level	Influencing Factor		
	A: NaCl (mmol/L)	B: Tris HCl (mmol/L)	C: Revolution Speed (*g*)
1	40	5	6000
2	50	10	10,000
3	60	20	14,000

**Table 2:** RPA reaction primer sequences of different animal-derived components.

Classification	Name	Sequences (5′ → 3′)	Amplified Fragment Length
Chicken	JI1F	CCTATGACAATAGAAGAATCAATGCTAAAATG	403 bp
	JI1R	TAATTTCATAGATTACCTACAGGAGACAGTTA	
	JI2F	GGTCTTAACTGTCTCCTGTAGGTAATCTATGA	276 bp
	JI2R	ATATTGGGTCTGGTTACTGTTGGTACTTTG	
	JI3F	CCTTAATCATCATCCAACCATTCATCATCC	362 bp
	JI3R	TTTAGGCAGTCATAGGTGTAGTCCGTATAG	
Beef	NIU1F	CTAACAATATACCAATGATGACGAGATGTTAT	111 bp
	NIU1R	GATAATAAAAAGAATTATTCCATAACGGAGGC	
	NIU2F	TACCAATGATGACGAGATGTTATCCGAGAA	370 bp
	NIU2R	CTTGTAGTAGTGTGAAGTAGACTCCTAATGTG	
	NIU3F	TGGCAGTCTCGCACTAACAG	435 bp
	NIU3R	AAGGCGTTTGAGGGGTAGTG	
Pork	ZHU1F	GAACTTTAACAGGCATCTGGTTCTTACTTC	221 bp
	ZHU1R	GTCCAGCTACAATTGATTTGACTGTGTTAG	
	ZHU2F	GATGAACTTTAACAGGCATCTGGTTCTTAC	224 bp
	ZHU2R	GTCCAGCTACAATTGATTTGACTGTGTTAG	
	ZHU3F	TGAAACCAGCAACCCGCTT	333 bp
	ZHU3R	TTGTTTGGATTGTCGTGCC	
Duck	YA1F	AACATGACCTAAATTTATTAGAGAAACTCC	230 bp
	YA1R	CATGTATATGTCTAGCAAAAACCAACTGTAAG	
	YA2F	CCATAATGATGAATGCTTGACAGACATACC	482 bp
	YA2R	CATATACGCCAACCGTCTCATTGAGTAATC	
	YA3F	CAACCAGAACAAGGCCCCA	216 bp
	YA3R	AAAATGTGAGGAGGGCGAGG	

**Table 3:** Purity and concentration of adulterated model DNA extracted using method D.

Data	Adulteration Model Ratio			
	1:1	1:5	1:10	1:20
*A*_260nm_/*A*_280nm_	1.547 ± 0.004	1.582 ± 0.002	1.610 ± 0.001	1.649 ± 0.002
DNA concentration (ng/µL)	264.2 ± 4.2	259.1 ± 7.1	274.8 ± 6.3	277.8 ± 5.8

## Data Availability

The original contributions presented in the study are included in the article/Supplementary Material, further inquiries can be directed to the corresponding author.

## Supplementary Materials

The following supporting information can be downloaded at: https://www.mdpi.com/article/10.3390/foods14233992/s1, Figure S1: Fluorescence reaction to assess the stability of the RPA detection system using SYBR Green I (1: active control 1d; 2: active control 2d; 3: active control 3d; 4: negative control 1d; 5: negative control 2d; and 6: negative control 3d), Table S1: PCR amplification reaction system, Table S2: Primer sequence of PCR reaction, Table S3: RPA amplification reaction system, Table S4: Concentration of DNA extracted using different methods, Table S5: Purity of DNA extracted using different methods, Table S6: Purity and concentration of DNA extracted using different pretreatment methods, Table S7: Orthogonal experimental design table of DNA extraction method, Table S8: Quantification of fluorescence G/R ratios during RPA time-course amplification, Table S9: Specificity assessment of RPA primers across adulteration models, Table S10: Stability assessment of the RPA method, Table S11: Sensitivity of the RPA method, Table S12: Summary of Method-Validation Metrics.
