# Supplementary material for: Rapid Detection of Animal-Derived Components in Plant-Based Meat Alternatives Using Recombinase Polymerase Amplification
Source: Foods. 2025 Nov 21;14(23):3992. doi: 10.3390/foods14233992 (PMC12692603; doi:10.3390/foods14233992)
Supplement: Supplementary file 1 [file foods-14-03992-s001.zip › foods-3960701-supplementary.pdf]

Supplementary Figure S1. Fluorescence reaction to assess the stability of the RPA detection system using SYBR Green I (1: active control 1d; 2: active control 2d; 3: active control 3d; 4: negative control 1d; 5: negative control 2d; and 6: negative control 3d).

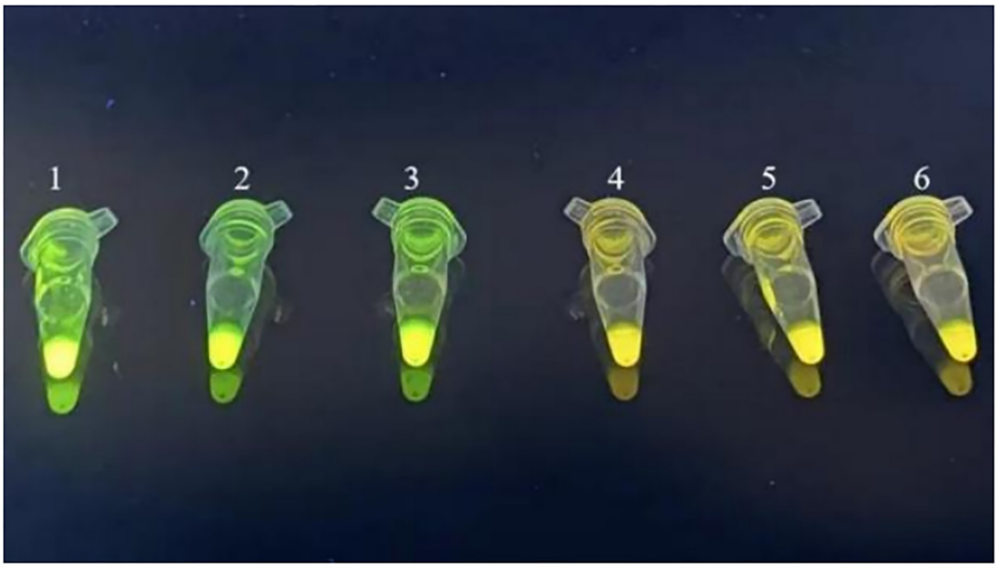

Supplementary Table S1. PCR amplification reaction system

Components	Volume（μL）
DNA Template	4
Upstream Primer	0.5
Downstream Primer	0.5
Premix	10
ddH_2O	5
Total volume	20

Supplementary Table S2. Primer sequence of PCR reaction

Primer	Sequences（5'→3'）	Amplified fragment length (bp)
16SrF	CTCGATGTTGGATCAGGACA	~119
16SrR	AGATAGAAACCGACCTGGATT	

Supplementary Table S3. RPA amplification reaction system

Component	Volume（μL）
A buffer	29.4
Upstream Primer（10μmol/L）	2
Downstream Primer（10μmol/L）	2
DNA Template	5
ddH_2O	9.1
B buffer	2.5
Total Volume	50

Supplementary Table S4. Concentration of DNA extracted using different methods

Sample	DNA concentration (ng/μL)				
	Method A	Method B	Method C	Method D	Method E
ZWC	247.2 ± 10.3^{d}	259.7 ± 7.2^{d}	309.8 ± 12.1^{a}	269.7 ± 4.2^{c}	283.9 ± 6.2^{b}
ZWB	234.6 ± 1.9^{e}	294.3 ± 4.8^{b}	298.4 ± 2.4^{a}	256.7 ± 3.7^{d}	273.6 ± 1.2^{c}
ZWP	263.6 ± 6.5^{b}	278.1 ± 2.3^{a}	279.4 ± 8.3^{a}	274.2 ± 5.2^{a}	278.7 ± 6.1^{a}

Note: Different lowercase letters indicate significant differences ($P < 0.05$) between different pretreatment methods for the same PMAs.

Supplementary Table S5. Purity of DNA extracted using different methods

Sample	A_{260nm}/A_{280nm}				
	Method A	Method B	Method C	Method D	Method E
ZWC	1.481 ± 0.004^{e}	1.496 ± 0.005^{d}	1.624 ± 0.006^{c}	1.653 ± 0.003^{b}	1.734 ± 0.009^{a}
ZWB	1.447 ± 0.002^{e}	1.543 ± 0.001^{d}	1.601 ± 0.007^{c}	1.637 ± 0.005^{b}	1.664 ± 0.004^{a}
ZWP	1.401 ± 0.007^{d}	1.569 ± 0.004^{c}	1.578 ± 0.005^{b}	1.565 ± 0.003^{c}	1.685 ± 0.001^{a}

Note: Different lowercase letters indicate significant differences ($P < 0.05$) between different pretreatment methods for the same PMAs.

Supplementary Table S6. Purity and concentration of DNA extracted using different pretreatment methods

Sample	Pretreatment method 1		Pretreatment method 2	
	A_{260nm}/A_{280nm}	DNA concentration (ng/μL)	A_{260nm}/A_{280nm}	DNA concentration (ng/μL)
ZWC	1.649 ± 0.006	297.2 ± 4.2	1.672 ± 0.005	310.4 ± 5.3
ZWB	1.642 ± 0.004	291.4 ± 3.1	1.663 ± 0.001	301.8 ± 1.4
ZWP	1.653 ± 0.001	301.6 ± 0.9	1.661 ± 0.003	318.2 ± 4.6

Supplementary Table S7. Orthogonal experimental design table of DNA extraction method

Experiment number	Influence factor			$A_{260\ nm}/A_{280\ nm}$
	NaCl	Tris CL	Centrifugal speed	
1	A_1	B_1	C_1	1.402±0.001
2	A_1	B_1	C_1	1.491±0.004
3	A_1	B_1	C_1	1.528±0.003
4	A_2	B_2	C_2	1.542±0.003
5	A_2	B_2	C_2	1.649±0.005
6	A_2	B_2	C_2	1.491±0.004
7	A_3	B_3	C_3	1.561±0.007
8	A_3	B_3	C_3	1.617±0.002
9	A_3	B_3	C_3	1.514±0.005
k_1	1.474	1.502	1.469	
k_2	1.561	1.586	1.550	
k_3	1.564	1.511	1.579	
Range	0.090	0.084	0.110	

Supplementary Table S8. Quantification of fluorescence G/R ratios during RPA time-course amplification

Sample	Time (min)	G/R Ratio (Mean ± SD)
A1	15	0.9948 ± 0.0121
A2	20	1.1219 ± 0.0065
A3	25	1.1572 ± 0.0082
A4	30	1.1799 ± 0.0095
A5	35	1.1924 ± 0.0073
B1	15	0.9882 ± 0.0142
B2	20	1.0974 ± 0.0104
B3	25	1.1001 ± 0.0088
B4	30	1.1473 ± 0.0116
B5	35	1.1524 ± 0.0091
C1	15	0.9833 ± 0.0137
C2	20	0.9957 ± 0.0123
C3	25	1.1443 ± 0.0105
C4	30	1.1605 ± 0.0079
C5	35	1.1775 ± 0.0084
D1	15	0.9655 ± 0.0158
D2	20	0.9551 ± 0.0146
D3	25	1.0149 ± 0.0112
D4	30	1.0326 ± 0.0127
D5	35	1.0824 ± 0.0096

Supplementary Table S9. Specificity assessment of RPA primers across adulteration models

Sample	Template Type	G/R Ratio (Mean ± SD)
A1	ZRC Adulteration Model	1.0721 ± 0.0083
A2	Soybean DNA	0.9883 ± 0.0105
A3	Blank (ddH$_2$O)	0.9987 ± 0.0091
B1	ZRB Adulteration Model	1.0875 ± 0.0076
B2	Soybean DNA	0.9632 ± 0.0112
B3	Blank (ddH$_2$O)	0.9395 ± 0.0128
C1	ZRP Adulteration Model	1.0586 ± 0.0089
C2	Soybean DNA	0.9634 ± 0.0107
C3	Blank (ddH$_2$O)	0.9471 ± 0.0115
D1	ZRD Adulteration Model	1.0719 ± 0.0085
D2	Soybean DNA	0.9672 ± 0.0109
D3	Blank (ddH$_2$O)	0.9825 ± 0.0098

Supplementary Table S10. Stability assessment of the RPA method

Sample	Storage Time (Day)	Template Type	G/R Ratio (Mean ± SD)	Statistical Significance
1	1	Beef Adulteration Model	1.0167 ± 0.0058	$p < 0.05$
2	2	Beef Adulteration Model	1.0179 ± 0.0062	$p < 0.05$
3	3	Beef Adulteration Model	1.0148 ± 0.0055	$p < 0.05$
4	1	Soybean DNA	0.9679 ± 0.0083	-
5	2	Soybean DNA	0.9628 ± 0.0091	-
6	3	Soybean DNA	0.9486 ± 0.0113	-

Note: Statistical analysis (one-way ANOVA followed by Duncan's test) revealed significant differences ($p < 0.05$) between the beef adulteration model and soybean DNA control groups across all three days of testing.

Supplementary Table S11. Sensitivity of the RPA method

Sample	DNA Concentration (ng/μL)	Detection Condition	G/R Ratio (Mean ± SD)
A1	300	365 nm UV Light	1.2448 ± 0.0132
A2	30	365 nm UV Light	1.2235 ± 0.0128
A3	3	365 nm UV Light	1.2146 ± 0.0145
A4	0.3	365 nm UV Light	0.9873 ± 0.0156
A5	0.03	365 nm UV Light	0.9582 ± 0.0163
A6	0 (Blank)	365 nm UV Light	0.9270 ± 0.0148
B1	300	Natural Light	1.0434 ± 0.0121
B2	30	Natural Light	1.0205 ± 0.0137
B3	3	Natural Light	0.7448 ± 0.0172
B4	0.3	Natural Light	0.8252 ± 0.0159
B5	0.03	Natural Light	0.8384 ± 0.0165

Supplementary Table S12. Summary of Method-Validation Metrics

Metric	Value (95% Confidence Interval)	Calculation Basis
Repeatability (Intra-assay CV)	1.19%	Triplicate G/R measurements at LoD (3 ng/μL, Sample A3)
Intermediate Precision (Inter-day CV)	0.16%	G/R ratios of the beef model over three independent days (n=3)
Sensitivity (Se)	100% (76.8% - 100%)	TP = 14, FN = 0
Specificity (Sp)	100% (73.8% - 100%)	TN = 12, FP = 0
Positive Predictive Value (PPV)	100% (76.8% - 100%)	TP = 14, FP = 0
Negative Predictive Value (NPV)	100% (73.8% - 100%)	TN = 12, FN = 0
